# 18-fluorodeoxyglucose positron emission tomography for tuberculosis diagnosis and management: a case series

**DOI:** 10.1186/1471-2466-13-14

**Published:** 2013-03-21

**Authors:** Scott K Heysell, Tania A Thomas, Costi D Sifri, Patrice K Rehm, Eric R Houpt

**Affiliations:** 1 Division of Infectious Diseases and International Health, University of Virginia, PO Box 801337, Charlottesville, VA 29908-1337, USA; 2 Office of Hospital Epidemiology/Infection Prevention and Control, University of Virginia Health System, Charlottesville, USA; 3 Department of Radiology and Medical Imaging, University of Virginia, Charlottesville, USA

**Keywords:** Positron emission tomography, Tuberculosis, Multidrug-resistant tuberculosis, Pulmonary nodule

## Abstract

**Background:**

F-fluorodeoxyglucose positron emission tomography (FDG-PET) is increasingly used to investigate for malignancy in the evaluation of pulmonary nodules, yet both active tuberculosis (TB) and malignancy have high uptake of FDG. Definitive diagnosis of TB can be further hindered in patients without growth of the organism from sputum.

**Case presentations:**

We describe a series of four representative cases of TB in varying disease state originally imaged by FDG-PET during evaluation for malignancy. Decisions regarding treatment for active TB in the presence of negative cultures and the evolving understanding of the spectrum of the TB disease state are discussed.

**Conclusions:**

FDG-PET may possess a role in the diagnosis of active TB infection in settings where conventional microbiological methods are unavaiable and holds particular promise for monitoring response to therapy in cases of unsettled treatment duration such as multidrug-resistant TB or in extrapulmonary TB.

## Background

Diagnosis of tuberculosis (TB) can be challenging in patients without growth of *Mycobacterium tuberculosis* in sputum or in patients with atypical extrapulmonary presentations. Tuberculin skin tests (TST) or serum interferon-gamma release assays can determine TB exposure but cannot distinguish between latent and active disease. Chest x-rays are performed as part of traditional evaluation for active disease, but in the modern era many individuals undergo chest computed tomography (CT) scans which reveal significantly more abnormalities. A common scenario is the patient with a pulmonary nodule diagnosed by CT, who is then referred for ^18^F-fluorodeoxyglucose positron emission tomography (FDG-PET), most commonly combined FDG-PET/CT, in order to evaluate for malignancy [1]. Yet, when imaged by FDG-PET, both malignant lesions and those with active *M. tuberculosis* infection may have high uptake of FDG [2]. We describe four challenging but representative cases evaluated for active pulmonary TB based on FDG-PET/CT performed for the work-up of malignancy and explore the potential niche for FDG-PET in TB management. Age, gender, country of origin and other identifiers have been removed for confidentiality, and cases were obtained from across the state.

## Case presentations

### Case 1

**Figure 1:**
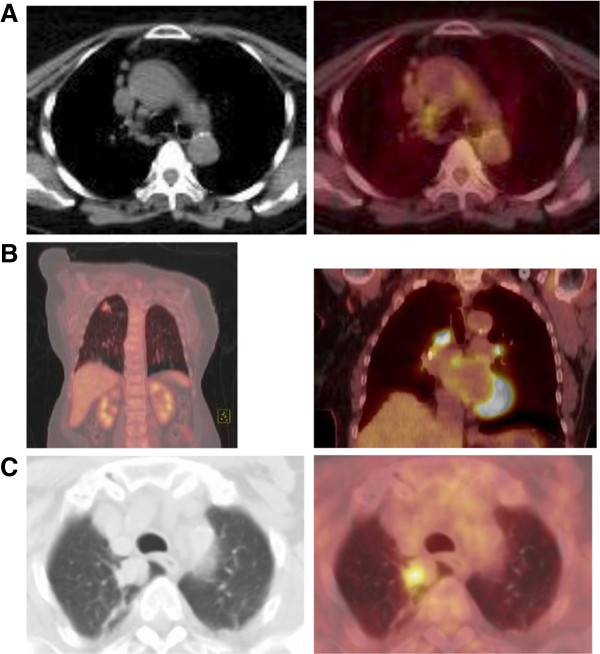
**Representative FDG-PET/CT images. A.** Case 1: axial CT (left) and fused FDG-PET/CT, with abnormal FDG uptake in the mediastinal lymph nodes. **B.** Case 2: fused FDG-PET/CT highlighting abnormal uptake in the right upper lobe nodule and more intense uptake in mediastinal lymph nodes. **C.** Case 3: axial CT (left) and fused FDG-PET/CT (right) with abnormal uptake in the right upper lobe nodule.

An elderly patient was born in a country non-endemic for TB, but had been exposed to active TB as a teenager and despite a positive TST, had never received latent TB infection treatment. The patient presented with 1–2 months of fatigue and cough. Radiograph revealed right and left upper lobe nodules with hilar and mediastinal lymphadenopathy. FDG-PET/CT scan found the left lung nodule with maximum SUV of 2.5 and a more intensely FDG-avid adjacent lymph node with maximum SUV of 5.1 (Figure 1A). Smear microscopy for acid-fast bacilli and mycobacterial culture of the sputum were negative. Ultimately a videoscopic wedge resection of the left lung found multiple granulomas and a mediastinal lymph node biopsy grew pan-susceptible *M. tuberculosis*. The patient was started on four-drug anti-tuberculosis therapy, but was intolerant of pyrazinamide, thus ultimately completed a total of 9 months of treatment with complete symptom and radiographic resolution. There was no evidence of disease recurrence at 1 year of follow-up after treatment completion.

### Case 2

An elderly patient emigrated from a TB endemic country five years prior and had known TB contacts, was found to have a poorly differentiated squamous cell carcinoma of the cervix. The patient endorsed fatigue, a non-productive cough and 10 pound weight loss in the antecedent 4 months. Pre-chemotherapy work-up included a positive TST. FDG-PET/CT was performed for cancer staging and demonstrated the cervix mass as well as an FDG avid, peripheral nodular lesion in the right upper lobe of the lung, measuring approximately 1.6 × 1.4 cm and prominent hypermetabolic mediastinal lymph nodes adjacent to the right upper lobe bronchus, the largest with a maximum standardized uptake value (SUV) of 11 (Figure 1B). Three sputum samples were negative for acid fast bacilli by smear microscopy. Given that the pulmonary imaging was atypical for metastatic cervical cancer and favored to represent TB infection, and that active TB could not be ruled out which carried significant patient care and infection control implications while hospitalized for urgent cancer therapy, the patient was started on 4-drug treatment for active pulmonary TB. After two months of therapy, excessive nausea precluded continuation of her TB treatment, all cultures remained negative, and thus no further therapy was immediately pursued. To date, the patient has not had return of TB symptoms, now greater than 6 months after conclusion of therapy.

### Case 3

An elderly patient from a non-endemic TB country was incidentally found to have a right upper lobe lung mass detected on a CT scan of the head/neck performed for evaluation of transient ischemic attacks. He was a lifetime non-smoker and had no symptoms of active TB. However, the patient was treated for TB as a young adult with 6 months of isoniazid and 4-aminobenzoic acid. To further evaluate the lung mass FDG-PET/CT scan of the chest was performed and revealed a hypermetabolic nodular lesion measuring 3.0 cm with a peak SUV of 5.75; no metabolically active perihilar or mediastinal lymph nodes were seen (Figure 1C). While a videoscopic wedge resection of the mass was negative for malignancy, gross pathology revealed a central cavity abutting the pleura. Histology found granulomatous inflammation and immunohistochemical staining for *M. tuberculosis* demonstrated rare intracellular rod forms. Bacterial, fungal and mycobacterial cultures as well as *M. tuberculosis* PCR of the tissue were negative. Given the histopathology and FDG-PET findings, anti-tuberculosis treatment was recommended but the patient refused and was lost to follow-up.

### Case 4

An elderly patient from a TB endemic country who had been treated for active TB 50 years prior, presented with fever and cough of one week’s duration following an international flight from the patient’s country of origin. Chest x-ray revealed biapical pleural thickening, bronchiectasis and a patchy right hilar consolidation. Expectorated sputum samples and bronchioalveolar lavage were unremarkable and negative for acid fast bacilli. The patient was planning on returning to the home country by plane as soon as medically cleared. The patient also had a history of multiple urological malignancies. In this setting FDG-PET/CT was performed which confirmed the biapical thickening and linear densities of the apices, but was without any significant FDG uptake in the lungs or adjacent lymph nodes. He was discharged without TB treatment and all cultures finalized as negative.

## Conclusions

FDG-PET/CT is an increasingly accessed technology for evaluation of malignancy in areas of both high and low TB prevalence [3] and therefore understanding the potential impact for its use in TB diagnosis and management is important for clinicians ordering and interpreting FDG-PET/CT. FDG uptake reflects cell glycolysis and is found in activated macrophages and lymphocytes [4], both of which are prominent in TB and other granulomatous inflammatory processes, as well as in neoplastic cells. The rate of uptake is reported as a standardized uptake value (SUV), the regional radioactivity concentration divided by the total injected dose and adjusted to the patient weight [5]. The maximum SUV is elevated in active TB. For instance, in a study of 150 subjects undergoing work-up for pulmonary nodules at a South Korean hospital, 10 cases of active pulmonary TB were identified and 9 (90%) had nodules with a maximum SUV above the threshold of 2.5 [6]. In a separate study of 25 subjects with culture-confirmed pulmonary TB, the mean maximum SUV was 4.96 ± 1.61 [7]. In addition, the maximum SUV declines on treatment, as demonstrated by a series of 21 HIV uninfected TB patients in whom the maximum SUV decreased by a median of 31% at one month of therapy [8]. Extrapulmonary sites of disease, particularly lymph nodes [9], may have an even higher maximum SUV, as was observed in Case 2. While quantitative assessment of SUV cannot distinguish active tuberculosis from malignancy [10], use of adjunctive tracers such as ^11^C-choline and ^18^F-fluorothymidine may provide improved discrimination. In a study of 97 patients with lung cancer, 14 with active pulmonary TB and 5 with non-tuberculous mycobacteria, the SUV of ^11^C-choline was low in active TB compared to lung cancer, and values of FDG and ^11^C-choline were both low in the patients with non-tuberculous mycobacteria [5].

Studies in humans examining FDG-PET for distinguishing latent and active TB are limited, but in one report of 25 patients, pulmonary TB disease activity was scored based on histopathology, culture and response to anti-tuberculosis treatment [11]. FDG-PET/CT revealed a significantly higher early and late phase SUV (60 and 120 minutes post-injection respectively) in patients with active compared to inactive TB [11]. Therefore a pulmonary nodule with high uptake on FDG-PET in a patient with evidence of TB exposure by positive TST or interferon-gamma release assay may tip the balance of treatment toward active TB, particularly if resection with histopathology and culture cannot be performed or if further immunosuppressing chemotherapy is immediately planned, as in Case 2.

**Figure 2:**
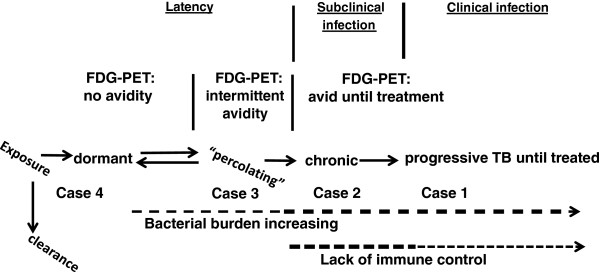
**Spectrum of tuberculosis infection with proposed relationship to FDG-PET findings.** Following exposure to *M. tuberculosis* an unknown percentage will clear infection entirely, while the vast majority develop latency (adapted from [10], [11]). Treatment of TB may eradicate all bacteria or leave a small population in a dormant non-replicating state. Populations with occasional replication termed “percolating” can be held under immunological control (noted by the dotted line) [10], [11]. Yet as bacterial burden increases, the risk of progression to active infection increases (noted by the hashed line). Cases 1–4 are presumptively placed along the proposed spectrum based on clinical, histopathological and microbiological studies and ^18^ F-fluorodeoxyglucose positron emission tomography (FDG-PET) avidity.

Complicating the matter, however, is recent work in non-human primates that suggests the TB disease state may be more dynamic than previously believed [12,13]. Rather than simply latent or active, TB infection occupies a more diverse spectrum (Figure 2). In certain individuals a robust acquired immune response with T-cell priming may have eliminated infection completely while in others a quiescent infection remains with some mycobacteria persisting in non-replicating form [13,14]. In a small proportion of patients, the immune response has kept active infection of replicating mycobacteria at the sub-clinical level, while in other patients, active inflammation, TB replication, and symptoms propagate [13], [14]. Therefore, high uptake of FDG by PET may represent ongoing active disease, as demonstrated by Case 1, or simply the host immune system activity that will ultimately prevail, such as could be argued for Case 3 (Figure 2). Given such emerging evidence from non-human primates, we would not advocate for the immediate adoption of FDG-PET/CT in treatment decisions regarding TB latency. However the implications of FDG-PET/CT findings must be considered in patients for whom this modality was employed for another clinical indication, as exemplified by Case 2, in whom low-grade TB symptoms may have been masked by the concurrent malignancy.

Perhaps the most promising role of FDG-PET/CT is in the monitoring of therapeutic response in select cases of extrapulmonary TB where sputum culture is unavailable, or in multidrug-resistant (MDR)-TB where second-line drugs are less efficacious and definitive treatment duration is not known [15]. Extrapulmonary tuberculoma may persist with little change for years if imaged with conventional radiology [16]. Uncontrolled case series have described the benefit of decreased FDG uptake following treatment in extrapulmonary sites of disease. In a series of 30 patients with TB of the spine for instance, the percent change in maximum SUV discriminated residual infection from successful treatment [17]. Another conceivable issue is that if imaged too early in the course of treatment, a paradoxical increase in FDG uptake may be observed due to immune reconstitution, however, this has not been observed in animal models [14]. The use of FDG-PET/CT has only begun to be reported for MDR-TB [18], but in a murine model of TB treatment FDG uptake correlated with mycobacterial burden in the lung and decreased proportional to the degree of bactericidal activity of the drug regimen [19]. Furthermore, in a promising series of HIV co-infected TB patients from South Africa, the number of lymph node bastions was found predictive of response to drug-susceptible TB treatment and a surrogate marker for clinical MDR-TB [18]. Given that the cost of treating MDR-TB may be 100 times more expensive than drug-susceptible TB, the decision to extend or shorten treatment, in some cases upward of 24 months, may defray the cost of FDG-PET/CT in certain well-resourced settings. Despite conceptual appeal for the use of FDG-PET/CT in assessment of transmissibility or even relapse following therapy, as was done in part for Case 4, further data are required to support these applications.

In summary, FDG-PET/CT is an increasingly employed modality and TB clinicians should become familiar with its use. FDG-PET/CT may need to be interpreted in the context of distinguishing latent versus active disease, and it has the potential to become a tool in monitoring treatment response in select cases of extrapulmonary TB or multidrug-resistance.

## Consent

The Institutional Review Board at the University of Virginia approved this report.

## Abbreviations

TB: Tuberculosis; TST: Tuberculin skin test; CT: Computed tomography; FDG-PET: ^18^ F-fluorodeoxyglucose positron emission tomography; SUV: Standardized uptake value.

## Competing interests

The authors declare that they have no competing interest.

## Authors’ contributions

SKH conceived of the study, performed literature review and analysis, and drafted the manuscript. TAT participated in chart review and provided critical revision of the manuscript. CDS cared for cases, provided oversight of infection control, interpretation of the literature and critical revision of the manuscript. PKR analyzed the imaging and provided critical revision of the manuscript. ERH cared for cases, conceived of the study, and provided critical revision of the manuscript. All authors read and approved the final manuscript.

## Pre-publication history

The pre-publication history for this paper can be accessed here:

http://www.biomedcentral.com/1471-2466/13/14/prepub
